# Nonsteroidal Anti‐Inflammatory Drug Prescriptions Are Associated With Increased Stress Fracture Diagnosis in the US Army Population

**DOI:** 10.1002/jbmr.3616

**Published:** 2018-12-10

**Authors:** Julie M Hughes, Craig J McKinnon, Kathryn M Taylor, Joseph R Kardouni, Lakmini Bulathsinhala, Katelyn I Guerriere, Kristin L Popp, Mary L Bouxsein, Susan P Proctor, Ronald W Matheny

**Affiliations:** ^1^ Military Performance Division United States Army Research Institute of Environmental Medicine Natick MA USA; ^2^ Department of Environmental Health Harvard T.H. School of Public Health Boston MA USA; ^3^ Endocrine Unit Massachusetts General Hospital Boston MA USA; ^4^ Center for Advanced Orthopedic Studies Beth Israel Deaconess Medical Center Boston MA USA; ^5^ Department of Orthopaedic Surgery Harvard Medical School Boston MA USA; ^6^ Research Service VA Boston Healthcare System Boston MA USA; ^7^ Department of Environmental Health Boston University School of Public Health Boston MA USA

**Keywords:** STRESS FRACTURE, NONSTEROIDAL ANTI‐INFLAMMATORY DRUGS (NSAID), IBUPROFEN, BONE FORMATION

## Abstract

Stress fractures are common in military personnel and endurance athletes, and nonsteroidal anti‐inflammatory drug (NSAID) use is widespread in these populations. NSAIDs inhibit prostaglandin synthesis, which blunts the anabolic response of bone to physical activity and could therefore increase risk of stress fracture. The objective of this study was to determine whether prescribed NSAIDs were associated with stress fracture diagnoses among US Army soldiers. We also aimed to establish whether acetaminophen, an analgesic alternative to NSAIDs, was associated with stress fracture risk. A nested case‐control study was conducted using data from the Total Army Injury and Health Outcomes Database from 2002 to 2011 (*n* = 1,260,168). We identified soldiers with a diagnosis of stress fracture (*n* = 24,146) and selected 4 controls per case matched on length of military service (*n* = 96,584). We identified NSAID and acetaminophen prescriptions 180 to 30 days before injury (or match date). We also identified soldiers who participated in basic combat training (BCT), a 10‐week period of heightened physical activity at the onset of Army service. Among these individuals, we identified 9088 cases and 36,878 matched controls. Conditional logistic regression was used to calculate incident rate ratios (RR) for stress fracture with adjustment for sex. NSAID prescription was associated with a 2.9‐fold increase (RR = 2.9, 95% confidence interval [CI] 2.8–2.9) and acetaminophen prescription with a 2.1‐fold increase (RR = 2.1, 95% CI 2.0–2.2) in stress fracture risk within the total Army population. The risk was more than 5‐fold greater in soldiers prescribed NSAIDs (RR = 5.3, 95% CI 4.9–5.7) and more than 4‐fold greater in soldiers prescribed acetaminophen (RR = 4.4, 95% CI 3.9–4.9) during BCT. Our results reveal an association between NSAID and acetaminophen prescriptions and stress fracture risk, particularly during periods of heightened physical activity. Prospective observational studies and randomized controlled trials are needed to support these findings before clinical recommendations can be made. © 2018 The Authors. *Journal of Bone and Mineral Research* published by Wiley Periodicals, Inc. on behalf of American Society for Bone and Mineral Research (ASBMR).

## Introduction

Stress fractures commonly occur in endurance athletes and military personnel, particularly during periods of heightened physical training. During military training, stress fractures are the second‐most common injury among women and the fifth‐most common injury among men, affecting up to 21% and 5% of female and male recruits, respectively.^1^, ^2^ It has been estimated that stress fractures cost the US Department of Defense approximately $100 million annually.^3^

Nonsteroidal anti‐inflammatory drugs (NSAIDs) such as ibuprofen and naproxen are commonly prescribed for their analgesic and anti‐inflammatory properties, and their use is widespread in the general,^4^ athletic,^5^ and military populations.^6^ In the US, 12% of adults reported taking NSAIDs at least 3 days per week for more than 3 months,^7^ and in the US Army, more than 80% of soldiers filled at least one NSAID prescription annually.^6^

With widespread use of NSAIDs, concern for potential adverse effects has been raised,^6^ such as gastrointestinal pain,^8^ renal and cardiovascular dysfunction,^9^, ^10^ and impaired post‐exercise skeletal muscle repair.^11^ Impaired fracture healing with NSAID use has also been documented^12^, ^13^, ^14^, ^15^ and has been partially attributed to anti‐prostaglandin activity of NSAIDs.^16^ NSAIDs inhibit cyclooxygenase (COX), an enzyme that catalyzes the conversion of arachidonic acid to prostaglandins and thromboxanes. Prostaglandin production is important not only for bone healing but also is an essential mediator of mechanically induced bone formation.^17^, ^18^, ^19^ This new bone formation confers a significant protective increase in skeletal fatigue resistance.^20^ Suppression of the adaptive bone formation response with NSAID use has been experimentally demonstrated in animals^(17–19)^ and provides biological plausibility for increased stress fracture risk.^21^, ^22^ Nevertheless, the relationship between NSAID use and stress fracture risk in humans is unknown. Accordingly, we examined the relationship between NSAID prescription and stress fracture risk among the entire US Active Duty Army population, hypothesizing that NSAID prescription would be associated with increased risk of stress fracture diagnosis. We also examined the relationship between acetaminophen prescription and stress fracture risk. Although not classified as an NSAID, acetaminophen possesses a mechanism of action distinct from that of NSAIDs and is commonly prescribed as a substitute for NSAIDs. Given this, we hypothesized that stress fracture risk would be substantially reduced or absent in soldiers prescribed acetaminophen compared with soldiers prescribed NSAIDs.

## Materials and Methods

### Study population

This study was approved by the Institutional Review Board at the US Army Research Institute of Environmental Medicine and complies with the World Medical Association Declaration of Helsinki—Ethical Principles for Medical Research Involving Human Subjects. A nested case‐control study was conducted within a cohort consisting of all US Army soldiers who were enlisted or commissioned between 2002 and 2011 (*n* = 1,260,168), using data extracted from the Total Army Injury and Health Outcomes Database (TAIHOD). The TAIHOD is a large repository of administrative (medical and personnel) data on the entire Active Duty Army population. It contains data from inpatient and outpatient health records on more than 6.5 million current and former soldiers that can be used for population‐level analyses.^23^, ^24^, ^25^, ^26^, ^27^

### Case definition

Incident stress fractures were identified using International Classification of Disease codes version 9 (ICD‐9). An incident case was defined as any soldier who received a diagnosis from the following list of ICD‐9 codes: 733.14, 733.15, 733.16, 733.93, 733.94, 733.95, 733.96, 733.97, and 733.98. The ICD‐9 codes 733.14 − 733.16 (pathological fractures) were included because before 2001, ICD‐9 codes for stress fracture did not exist, and military clinicians would commonly use pathological fracture codes.^28^, ^29^ For these analyses, a soldier was considered to be a case when he or she received either a diagnosis for stress fracture during an inpatient encounter or during two outpatient encounters separated by at least 14 days but less than 90 days.

### Control selection

Risk set matching was used to match each stress fracture case to 4 controls. Soldiers were eligible to be selected as a matched control if they were in the Army on the date of an identified case's injury. Controls were persons identified with no history of stress fracture and were matched to cases based on categorical length of time in the service (<4 months, 4 months to <1 year, 1 to <3 years, 3 to <5 years, and ≥5 years).

### Exposure definition

The main exposure was classified as receiving a prescription for NSAIDs, which included ibuprofen, naproxen, meloxicam, and indomethacin. These particular NSAIDs were chosen because they are the most frequently prescribed NSAIDs among soldiers.^6^

Individuals were classified as being an NSAID user if they were prescribed an NSAID 30 to 180 days before the date of a confirmed diagnosis of stress fracture if classified as a case, or before the date of matching if classified as a control. The timeframe of 30 to 180 days was selected as the window that allowed for avoidance of potential reverse causation while allowing for a biologically plausible window for the effect of NSAIDs on stress fracture onset.

Using this information, we created a binary variable (yes/no) for ever having received a prescription for any of the named NSAIDs or acetaminophen. A binary variable was also created for ever having received a prescription for any of the four individual NSAID prescriptions separately.

Because of the possibility of reverse causation (that individuals were prescribed NSAIDs after the onset of a stress fracture but before a diagnosis), we applied lags at 15, 30, or 45 days to the exposure, redefining who was an NSAID user at each lagged analysis. This excludes those persons who had an NSAID prescription within 15, 30, or 45 days before the first encounter for stress fracture for cases or before the match date for controls. After implementing these lags, it was decided that final models would utilize a 30‐day lag for the exposure definition, thus allowing for avoidance of capturing NSAID use that cannot contribute to the onset of the fracture, while also allowing for a sufficient window of time for NSAIDs to influence stress fracture risk.

### Covariates considered

A list of potential covariates was identified *a priori*, based on review of the literature on factors known to influence stress fracture risk. The covariates considered for our models were age, sex, race, ethnicity, education level, and body mass index (BMI). The latter two variables were measured at accession into the Army. Age was categorized as <20, 20 to 30, and >30 years old at the time of injury or matching; sex was male or female; race was defined as white, black, Asian, American Indian, or other; ethnicity was classified as Hispanic or non‐Hispanic; BMI was categorized as underweight (<18.5 kg/m^2^), normal (18.5 to 24.9 kg/m^2^), overweight (25.0 to 29.9 kg/m^2^), and obese (>30.0 kg/m^2^); and education was categorized as less than high school, high school, college, or attainment of an advanced degree. Variables were considered confounders and thus important to include in the final analytic model if they changed the relative risk of the association between stress fracture and NSAID prescription by ≥10% when examined individually.

### Data analyses

The frequency of descriptive and demographic characteristics of the case‐control groups was reported. Incident rate ratios (RR) and their corresponding 95% confidence intervals (CI) were estimated using a conditional logistic regression adjusting for potential confounders. Final models were stratified by sex to evaluate if there are sex‐specific differences for the association between NSAID use and stress fracture diagnoses. As a secondary analysis, effect modification was evaluated for race and BMI status on the effect of NSAIDs on stress fracture risk. This was done by adding an interaction term to the final model between NSAIDs and race and BMI, respectively, producing two separate models.

All regression models were evaluated in the nested case‐control population (“full Army population”) and in a subset of the population composed of individuals who had been in the military for less than 11 weeks. The 11‐week window was set to capture the time during basic combat training (BCT). BCT is a period at the beginning of military service that is characterized by increased levels of physical activity and high incidence of stress fracture.^30^, ^31^ All analyses were performed using SAS software (version 9.3; SAS Institute, Inc., Cary, NC, USA).

### Sensitivity analysis

To more conservatively address the potential for reverse causation, an additional analysis was conducted using the full Army population, restricting NSAID and acetaminophen users to those who were prescribed NSAIDs or acetaminophen for diagnoses unrelated to musculoskeletal injury or pain. RRs and 95% CIs were calculated using conditional logistic regression.

## Results

Table 1 presents demographic characteristics of the US Army study population from 2002 to 2011. The full Army population was primarily male (79.4%), white (75.8%), and between the ages of 20 and 30 (59.4%); this same demographic distribution was found among the subset of individuals in the Army during the initial 11‐week BCT period (BCT‐only subgroup).

**Table 1:** Characteristics of Army Study Population (2002–2011) by Stress Fracture Case Status

	Full Army population		In Army service <11 weeks (basic combat training)	
Variable	Stress fracture cases (*n* = 24,146) *n* (%)	Controls (*n* = 96,584) *n* (%)	Stress fracture cases (*n* = 9088) *n* (%)	Controls (*n* = 36,878) *n* (%)
Sex				
Male	14,203 (58.8)	81,608 (84.5)	5089 (56)	30,758 (83.4)
Female	9934 (41.2)	14940 (15.5)	3999 (44)	6120 (16.6)
Race				
White	18,748 (77.7)	72,733 (75.3)	7421 (81.7)	28,674 (77.8)
Black	3717 (15.4)	16,864 (17.5)	1134 (12.5)	5962 (16.2)
Asian	889 (3.7)	3542 (3.7)	369 (4.1)	1487 (4)
American Indian	267 (1.1)	910 (0.9)	109 (1.2)	378 (1)
Other	88 (0.4)	390 (0.4)	23 (0.3)	104 (0.3)
Unknown	428 (1.8)	2109 (2.2)	32 (0.4)	273 (0.7)
Ethnicity				
Hispanic	2675 (11.1)	10921 (11.3)	930 (10.2)	4227 (11.5)
Non‐Hispanic	21,175 (87.7)	84,259 (87.3)	8055 (88.6)	32,178 (87.3)
Unknown	287 (1.2)	1368 (1.4)	103 (1.1)	473 (1.3)
Age (years)				
<20	5832 (24.2)	29,626 (30.7)	2821 (31)	15,742 (42.7)
20–30	14,867 (61.6)	56,824 (58.9)	5420 (59.6)	19,719 (53.5)
>30	3432 (14.2)	10,079 (10.4)	843 (9.3)	1407 (3.8)
Unknown	6 (0)	19 (0)	4 (0)	10 (0)
Education				
Did not graduate high school	145 (0.6)	670 (0.7)	31 (0.3)	222 (0.6)
High school	15,741 (65.2)	64,009 (66.3)	5754 (63.3)	23,730 (64.3)
College	7409 (30.7)	26,371 (27.3)	3022 (33.3)	10,114 (27.4)
Advanced degree	376 (1.6)	1803 (1.9)	55 (0.6)	302 (0.8)
Unknown	466 (1.9)	3695 (3.8)	226 (2.5)	2510 (6.8)
Body mass index (kg/m^2^)				
<18.5	773 (3.2)	1870 (1.9)	390 (4.3)	770 (2.1)
18.5–24.9	12,855 (53.3)	46,033 (47.7)	5188 (57.1)	18,576 (50.4)
25.0–29.9	7976 (33)	34,355 (35.6)	2826 (31.1)	12,742 (34.6)
>30	1895 (7.9)	9734 (10.1)	616 (6.8)	3700 (10)
Unknown	638 (2.6)	4556 (4.7)	68 (0.7)	1090 (3)

From 2002 through 2011, a total of 24,146 stress fracture cases were diagnosed among the full Army population, with 5.5% of those cases coded as pathological fractures. Within the BCT‐only population, 9088 stress fractures were identified. When compared with the control population, a greater proportion of stress fracture cases were observed in women. Of the stress fractures identified in the full Army population, 41.6% were not classified with a specific location of injury. Of the 58.4% that were classified by location, 9.6% were at the femoral neck, 2.8% at the femoral shaft, 63.3% at the tibia or fibula, 19.4% at the metatarsals, and 4.9% at the pelvis.

We next identified the frequency of NSAID prescriptions in both the full Army population and in the BCT‐only subgroup. As shown in Table 2, ibuprofen and naproxen were prescribed most frequently among both stress fracture cases and controls. Meloxicam, indomethacin, and acetaminophen were also prescribed, although with less frequency than ibuprofen or naproxen.

**Table 2:** Description of NSAID Use in the Army (2002–2011) by Case Status

	Full Army population		In Army service <11 weeks (basic combat training)	
Prescription type	Stress fracture cases (*n* = 24,146) *n* (%)	Controls (*n* = 96,584) *n* (%)	Stress fracture cases (*n* = 9088) *n* (%)	Controls (*n* = 36,878) *n* (%)
Any NSAID	9978 (41.3)	18,622 (19.3)	1835 (20.2)	1505 (4.1)
Ibuprofen	7997 (33.1)	15,201 (15.7)	1557 (17.1)	1297 (3.5)
Naproxen	3796 (15.7)	5307 (5.5)	409 (4.5)	249 (0.7)
Indomethacin	519 (2.2)	645 (0.7)	59 (0.6)	37 (0.1)
Meloxicam	640 (2.7)	884 (0.9)	13 (0.1)	3 (0)
Acetaminophen	3499 (14.5)	7309 (7.6)	607 (6.7)	672 (1.8)

Next, we examined the degree to which NSAID prescription was associated with increased risk of stress fracture. As presented in Table 3 (Model 1a), NSAID prescription overall was associated with a 2.9‐fold (95% CI 2.8–2.9) increase in stress fracture risk within the full Army population, and a 5.3‐fold (95% CI 4.9–5.7) increase in stress fracture risk within the BCT‐only subgroup. When evaluating the individual effects of ibuprofen, naproxen, or indomethacin on the risk of stress fracture in the full Army population (Table 3, Model 1b), stress fracture risk remained statistically elevated with RRs ranging from 2.1 (indomethacin; 95% CI 1.8–2.3) to 2.6 (naproxen; 95% CI 2.5–2.7). Individuals in the BCT‐only subgroup possessed higher RRs than the full Army population for all NSAID types. Acetaminophen prescription was associated with increased stress fracture risk in both the full Army population and in the BCT‐only subgroup (Table 3, Model 2). Additionally, the BCT‐only group demonstrated a >2‐fold increased risk of stress fracture with acetaminophen prescription compared with the full Army population (RR = 4.4, 95% CI 3.9–4.9 for BCT‐only versus RR = 2.1, 95% CI 2.0–2.2 for the full Army population).

**Table 3:** Sex‐Adjusted Incident Rate Ratio (RR) for Stress Fracture by NSAID Usage (2002–2011)

Model	Prescription	RR (95% confidence interval [CI]) full Army population	RR (95% CI) basic combat training
Model 1a	All NSAIDs	2.9 (2.8–2.9)	5.3 (4.9–5.7)
Model 1b	Ibuprofen	2.2 (2.1–2.3)	4.5 (4.2–4.9)
	Naproxen	2.6 (2.5–2.7)	4.8 (4.1–5.8)
	Indomethacin	2.1 (1.8–2.3)	2.9 (1.8–4.7)
Model 2	Acetaminophen	2.1 (2.0–2.2)	4.4 (3.9–4.9)

All models are adjusted for sex.

Model 1a: Any NSAID prescription (yes/no) as a binary variable in the model.

Model 1b: Each individual NSAID in the model. Because of the limited number of persons with meloxicam prescriptions among individuals in the Army for less than 11 weeks, this NSAID type was not included in the analysis.

Model 2: Only acetaminophen (yes/no) as a binary variable in the model.

Sex‐stratified models revealed that both male and female soldiers with NSAID prescriptions had higher risk of stress fracture (RR = 3.0, 95% CI 2.9–3.1 and RR = 2.6, 95% CI 2.5–2.8, respectively). Additionally, the sex‐stratified RRs within the BCT‐only subgroup were higher than corresponding control (RR = 5.5, 95% CI 5.0–6.1 and RR = 4.8, 95% CI 4.2–5.5, for males and females, respectively).

As with NSAID prescription, acetaminophen prescriptions were associated with increased risk of stress fracture in both male and female soldiers when compared with their male and female counterparts not prescribed acetaminophen (RR = 4.6, 95% CI 4.0–5.2 and RR = 3.3, 95% CI 2.5–4.2, respectively).

After considering all potential confounders for the relationship between NSAIDs and stress fracture, final models were adjusted only for sex. Although there was a decrease in the number of cases included in the analysis (*n* = 23,235) due to covariate missingness, the results for the models adjusting for age, sex, race, ethnicity, education level, and BMI were comparable to the results found for the models that only adjusted for sex (Supplemental Table S1).

No effect modification existed by BMI status (*p* < 0.05), although there was effect modification on the relationship between NSAIDs and stress fracture risk by race/ethnicity. More specifically, using whites prescribed NSAIDs as a reference group, blacks prescribed NSAIDs possessed a 36% lower risk of stress fracture (RR = 0.64, 95% CI 0.25–0.72). There were no differences in risk between those prescribed NSAIDs in the other race/ethnicity categories analyzed (Hispanic and other) compared with whites prescribed NSAIDs. It is important to note that total risk of stress fracture among those prescribed NSAIDs was elevated across all races.

### Sensitivity analyses

To evaluate the presence of reverse causation, lagged analyses were performed. When consideration of NSAID and acetaminophen prescriptions was excluded within 15 and 45 (instead of 30) days before stress fracture for cases and before match date for the controls, the RR for stress fracture diminished with increasing lag time but did not lose significance across any lagged period (15‐day lag: RR = 3.9, 95% CI 3.7–4.0; 45‐day lag: RR = 2.4, 95% CI 2.3–2.5, Table 4). The largest decline in risk occurred between the 15‐day lag and 30‐day lag.

**Table 4:** Lagged Analysis Results Using 15‐, 30‐, and 45‐Day Lags From the Initial Stress Fracture Diagnosis

	Lag, rate ratio (95% confidence interval)		
Prescription type	15‐day lag	30‐day lag	45‐day lag
All NSAIDs	3.9 (3.7–4.0)	2.9 (2.8v2.9)	2.4 (2.3–2.5)
Ibuprofen	2.7 (2.6–2.8)	2.2 (2.1–2.3)	2.0 (1.9–2.0)
Naproxen	3.1 (2.9–3.2)	2.6 (2.5–2.7)	2.3 (2.2–2.4)
Indomethacin	2.5 (2.2–2.8)	2.1 (1.8–2.3)	2.0 (1.7–2.3)
Acetaminophen	2.3 (2.3–2.4)	2.1 (2.0–2.2)	1.9 (1.8–2.0)

All results are adjusted for sex.Table 5 Sex‐Adjusted Incident Rate Ratio (RR) for Stress Fracture by NSAID Prescription Among Persons Who Were Prescribed NSAIDs or Acetaminophen for Diagnoses Unrelated to Musculoskeletal Injury or Pain

When restricting the NSAID and acetaminophen user population to those who were prescribed these medications for reasons unrelated to musculoskeletal injury or pain (eg, acute upper respiratory, acute pharyngitis, other unknown and unspecified cause of morbidity, acute nasopharyngitis, tobacco use disorder, and acute bronchitis; see Supplemental Table S2), this sensitivity analysis still showed an elevated risk of stress fracture among NSAID users (RR = 1.74, 95% CI 1.66–1.82) and acetaminophen users (RR = 1.36, 95% CI 1.27–1.46) (Table 5).

**Table 5:** Sex‐Adjusted Incident Rate Ratio (RR) for Stress Fracture by NSAID Prescription Among Persons Who Were Prescribed NSAIDs or Acetaminophen for Diagnoses Unrelated to Musculoskeletal Injury or Pain

		Cases (*n* = 16,879)	Controls (*n* = 86,166)	
Model	Prescription	Prescription *n* (%)	Prescription *n* (%)	RR (95% confidence interval) full Army population
Model 1a	All NSAIDs	3529 (20.9)	11,492 (13.2)	1.74 (1.66–1.82)
Model 1b	Ibuprofen	2859 (16.9)	9493 (10.89)	1.61 (1.53–1.69)
	Naproxen	781 (4.6)	2043 (2.3)	1.89 (1.73–2.07)
	Indomethacin	86 (0.5)	255 (0.3)	1.50 (1.16–1.95)
Model 2	Acetaminophen	1627 (9.6)	5275 (6.1)	1.36 (1.27–1.46)

All models are adjusted for sex.

Model 1a: Any NSAID prescription (yes/no) as a binary variable in the model.

Model 1b: Each individual NSAID in the model. Because of the limited number of persons with meloxicam prescriptions among individuals in the Army for less than 11 weeks, this NSAID type was not included in the analysis.

Model 2: Only acetaminophen (yes/no) as a binary variable in the model.

## Discussion

Our study in a large cohort of Active Duty Army soldiers revealed that incidence of stress fracture diagnosis was elevated in both men and women prescribed NSAIDs or acetaminophen (nearly 3‐fold increased risk in soldiers prescribed NSAIDs and >2‐fold increase in risk in soldiers prescribed acetaminophen). When we focused our analyses to include only those individuals during the BCT period, a time when soldiers experience heightened physical training, we found a >4‐fold increase in stress fracture risk with NSAID and acetaminophen prescription.

The motivation to perform this study originated from reports of diminished mechanical loading‐induced bone anabolism after NSAID administration in humans^32^ and in some,^17^, ^19^, ^33^, ^34^, ^35^ but not all,^36^ studies in animals. Decreased bone anabolism was particularly evident when NSAIDs were administered before each loading bout in animal studies^17^, ^32^, ^34^ and when administered before, rather than after, each exercise bout in healthy premenopausal women undergoing a 36‐week exercise intervention.^32^

Suppression of mechanically induced bone anabolism when NSAIDs are administered before mechanical loading may be due to the role prostaglandins play as secondary messengers in bone cell mechanotransduction.^33^ Prostaglandin production is an early response to mechanical stimulation in osteoblasts and osteocytes^37^ and has been shown to stimulate osteoblast differentiation.^16^, ^38^ NSAIDs attenuate prostaglandin production and may therefore impair the anabolic response to mechanical loading by inhibiting COX‐1 or COX‐2, key enzymes involved in prostaglandin production.^37^ Attenuated bone formation after mechanical loading may, in turn, have important implications for stress fracture risk. Stress fractures commonly occur during periods of increased repetitive loading,^39^ when a robust anabolic bone response may confer critical mechanical advantages.^20^ Such mechanically stimulated bone formation improves the fatigue life of bone,^20^ and inhibition of bone formation with NSAID use during periods of increased physical activity could potentially mute these effects, thus predisposing to stress fracture.

NSAIDs are some of the most commonly used drugs in the world, and in the U., 12% of adults report taking aspirin or NSAIDs regularly.^7^ Within the general population, risk of stress fracture is greatest among physically active individuals such as endurance athletes.^39^, ^40^ In turn, use of NSAIDs is common among athletes, with 75% of student‐athletes reportedly taking NSAIDs regularly.^41^ Given the widespread use of NSAIDs in the general and athletic populations, and given our findings suggesting an elevated risk of stress fracture during the physically demanding period of BCT, prospective observational studies and randomized controlled trials should be performed to confirm whether there is an elevated risk of stress fracture in physically active populations who commonly use these drugs.

Contrary to our hypothesis, we found that stress fracture risk was increased in soldiers prescribed acetaminophen. Our hypothesis was based primarily on the premise that acetaminophen is known to possess a mechanism of action different from that of NSAIDs.^42^ However, like NSAIDS, acetaminophen has also been shown to inhibit COX and prostaglandin biosynthesis in various tissues, although the degree to which this inhibition is comparable to that exhibited by NSAIDs has not been consistent across all studies. For example, Schwartz and colleagues^43^ found that indomethacin repressed the PGI2 urinary metabolite PGI‐M to a greater extent than acetaminophen, though both drugs significantly inhibited PGI‐M compared with placebo. Likewise, various NSAIDs have been shown to repress lipopolysaccharide‐induced PGE2 production at concentrations orders of magnitude less than acetaminophen in cultured rat cerebral endothelial cells^44^ and in rat brain microglia cultures.^45^ Finally, acetaminophen had no effect on PGE2 levels in gingival crevicular fluid in subjects undergoing orthodontic tooth movement treatment, whereas PGE2 levels were significantly reduced in subjects administered ibuprofen.^46^

In contrast to these observations, there are reports that acetaminophen can inhibit prostaglandin production to a similar degree as NSAIDs at over‐the‐counter doses. For example, in skeletal muscle, exercise‐induced increases in PGE2 were significantly reduced in subjects that consumed acetaminophen but not in subjects that received ibuprofen or placebo.^47^ Additionally, acetaminophen reduced PGE2 production to the same degree as the COX‐2 inhibitor rofecoxib in transudate collected from the surgical site after molar extraction.^48^ Moreover, acetaminophen has been shown to inhibit COX‐2 in human whole blood and isolated monocytes, revealing anti‐inflammatory activity similar to that found with NSAID exposure.^49^

These studies demonstrate that acetaminophen may be less effective, or as effective, as NSAIDs in inhibiting prostaglandin production, depending on the tissue examined and the experimental model. Because acetaminophen has been shown to inhibit prostaglandin synthesis to at least some degree in various tissues in vivo, and because prostaglandins have been shown to mediate bone repair and mechanically induced bone formation, our finding that acetaminophen prescription was associated with stress fracture is generally consistent with published work. However, risk of stress fracture in soldiers prescribed acetaminophen was generally similar to that observed in soldiers prescribed other NSAIDs during BCT. Acetaminophen has been shown to possess less potency and specificity toward COX enzymes compared with other NSAIDs^50^ and would therefore be expected to reduce prostaglandin levels to a lesser degree than other NSAIDs. However, BCT involves multiple environmental, physical, and psychological stressors that, independently and in combination, may modify prostaglandin biosynthesis, stability, or signaling. Under these conditions, the extent to which acetaminophen or other NSAIDs impact prostaglandin levels is not known. Ultimately, prospective studies are necessary to substantiate these observations.

There are several limitations to this study. First, because we relied upon medical records for NSAID prescription data, we were dependent on intention‐to‐treat analysis (individuals were defined as NSAID users based on their prescription and not on whether they actually took the prescribed medication). Because the TAIHOD consists of electronically compiled medical data and lacks data regarding non‐prescription NSAID use (ie, over‐the‐counter purchases of NSAIDs), it is possible that the soldiers we evaluated were consuming over‐the‐counter NSAIDs. However, soldiers attending BCT live in a controlled environment where medication can only be obtained with a prescription from a military clinician and compliance is tightly regulated. Any medication, including analgesics brought to BCT, are turned in upon arrival to the military training base, with medication provided to the recruit only through the established medical system during this time period; thus, all NSAID usage during basic training should be associated with a recorded prescription. Although there exists increased potential for misclassification of NSAID use outside the BCT period because we cannot account for over‐the‐counter drug use, it is likely that non‐prescription use of NSAIDs in the BCT subgroup was minimal or absent.

On the other hand, soldiers that have graduated BCT and have begun their duties in their operational units do have access to over‐the‐counter medications. Soldiers perform their duties within occupational fields such as infantry, health care, or communications, and are deployed worldwide on all continents. Soldiers are exposed to friendly and hostile environments, and a day in the life of a particular soldier will depend on their job, geographic location, and whether they are engaged in active conflict. In almost all cases, soldiers have immediate access to some level of medical care, ranging from a single medical provider within a small Special Operations team to a fully equipped and staffed Army hospital. Although medical care, including provision of NSAIDs, is free to Active Duty soldiers, over‐the‐counter NSAID use will not be reflected within the TAIHOD and, thus, represents a limitation to this study.

Another potential limitation is that, because NSAIDs are prescribed for pain, the potential for reverse causation exists (that is, we cannot be sure that stress fracture cases with NSAID prescriptions were not prescribed NSAIDs to reduce pain associated with a preclinical stress fracture). In this study, we addressed this concern with a set of lagged analyses that examined the effects of NSAID prescription on stress fractures over differing time windows. These analyses presumably removed inclusion of NSAID exposure that occurred after the onset of a stress fracture. We also addressed the potential for reverse causation with a sensitivity analysis in which we restricted the NSAID and acetaminophen populations to those who were prescribed those medications for reasons unrelated to musculoskeletal injury or pain. From this analysis, we found that the association between elevated stress fracture risk and analgesic prescription remained. These results provide evidence that the relationship between analgesic prescriptions and stress fracture risk cannot be completely explained by reverse causation. Nonetheless, given the source and level of detail available in the analytic data, it is impossible to know precisely when the onset of the stress fracture occurred. Finally, although the strength of our database is in its size and completeness as an indicator of medical utilization, it lacks information on certain lifestyle factors, such as history of physical activity, which could potentially confound the relationship between NSAIDs and stress fracture. The database structure also lacks information about whether imaging was performed and whether, if done, stress fractures were confirmed by those imaging studies. Although this is a limitation of this study, we utilized a conservative definition of stress fracture in which an individual was classified as having a stress fracture if he or she received a diagnosis during an inpatient encounter or two outpatient encounters with 14 to 90 days separating the two visits. Although an inpatient visit likely signifies a more serious stress fracture injury, requiring at least two outpatient visits helps ensure that the analytic data set includes only those cases whose clinicians were able to make a second diagnosis after an earlier suspected stress fracture diagnosis. This approach likely reduces the number of cases who would be potentially misdiagnosed with a stress fracture during their first clinical visit.

In summary, we identified an approximately 3‐fold increased risk of diagnosed stress fracture in soldiers prescribed NSAIDs. There was an even greater risk of stress fracture in soldiers prescribed NSAIDs while participating in BCT, a period of heightened physical activity at the beginning of military service. These results suggest an association between NSAID prescription and stress fracture diagnoses in soldiers; however, prospective observational studies and randomized controlled trials are needed to support these findings before clinical recommendations can be made.

## Disclosures

All authors state that they have no conflicts of interest.

## Supporting information

Supporting Data.
