# Supplementary material for: Nonsteroidal Anti‐Inflammatory Drug Prescriptions Are Associated With Increased Stress Fracture Diagnosis in the US Army Population
Source: J Bone Miner Res. 2018 Dec 10;34(3):429–36. doi: 10.1002/jbmr.3616 (PMC6936225; doi:10.1002/jbmr.3616)
Supplement: Supplementary file 1 — Supporting Data. [file JBMR-34-429-s001.docx]

Supplemental Table 1: Adjusted RR for stress fracture by NSAID usage using a 30 day lag for the exposure. All models are adjusted for sex, age, race, ethnicity, education, ethnicity, and BMI and subjects were matched for length of service and date of injury.

| **Model** | **Prescription Type** | **RR (95% CI)**  **Full Army population** | **RR (95% CI)**  **In Army service <11 weeks**  **(Basic Combat Training)** |
| --- | --- | --- | --- |
| Model 1a | All NSAIDs | 2.8 (2.7,2.9) | 4.9 (4.6,5.3) |
| Model 1b | Ibuprofen | 2.1 (2.0,2.2) | 4.2 (3.9,4.6) |
|  | Naproxen | 2.6 (2.4,2.7) | 4.6 (3.8,5.5) |
|  | Indomethacin | 2.0 (1.7,2.2) | 2.5 (1.5,4.1) |
| Model 2 | Acetaminophen | 2.0 (1.9,2.1) | 4.2 (3.8,4.8) |

Model 1a: Any NSAID prescription (Yes/No) as a binary variable in the model.

Model 1b: Each individual NSAID in the model. Due to the limited number of persons with meloxicam prescriptions among individuals in the Army for less than 11 weeks, this NSAID type was not included in the analysis.

Model 2: Only acetaminophen (Yes/No) as a binary variable in the model.

RR= Risk Ratio

Supplemental Table 2: Top 20 diagnoses associated with NSAID prescriptions

| **Diagnosis** | **Percent** | **Count** |
| --- | --- | --- |
| Acute upper respiratory | 7.0 | 7711 |
| **Pain in joint, lower leg** | 6.0 | 6517 |
| **Pain in limb** | 4.1 | 4462 |
| **Sprain and strain of knee and leg** | 3.0 | 3243 |
| **Pain in joint, ankle and foot** | 2.8 | 3115 |
| Acute pharyngitis | 2.7 | 2957 |
| **Sprain of ankle** | 2.3 | 2480 |
| **Pain in joint, pelvic region and thigh** | 1.8 | 1968 |
| **Lumbago** | 1.8 | 1948 |
| Other unknown and unspecified cause of morbidity | 1.5 | 1692 |
| **Backache, unspecified** | 1.4 | 1502 |
| Acute nasopharyngitis | 1.4 | 1501 |
| Tobacco use disorder | 1.1 | 1227 |
| Acute bronchitis | 1.1 | 1182 |
| Screen for condition not elsewhere classified | 1.1 | 1155 |
| Ill-defined condition | 1.0 | 1054 |
| Headache | 0.9 | 1017 |
| Joint pain in shoulder | 0.9 | 992 |
| Physical therapy not elsewhere classified | 0.9 | 943 |
| **Plantar fascial fibromatosis** | 0.9 | 942 |

NOTE: **Bold** text in table = Diagnoses removed for the sensitivity analysis, due to possible association with present stress fracture
